# Complementary epitopes and favorable developability of monoclonal anti-LAMP1 antibodies generated using two transgenic animal platforms

**DOI:** 10.1371/journal.pone.0235815

**Published:** 2020-07-16

**Authors:** Beatrice Cameron, Tarik Dabdoubi, Laurence Berthou-Soulié, Marie Gagnaire, Isabelle Arnould, Anne Severac, Fabienne Soubrier, Jacqueline Morales, Philip A. Leighton, William Harriman, Kathryn Ching, Yasmina Abdiche, Katarina Radošević, Thomas Bouquin

**Affiliations:** 1 Biologics Research, Sanofi R&D, Boufféré, France; 2 Ligand Pharmaceuticals Inc., San Diego, California, United States of America; 3 Carterra Inc., Salt Lake City, Utah, United States of America; University of Saskatchewan, CANADA

## Abstract

Monoclonal antibodies (mAbs) for therapeutic applications should be as similar to native human antibodies as possible to minimize their immunogenicity in patients. Several transgenic animal platforms are available for the generation of fully human mAbs. Attributes such as specificity, efficacy and Chemistry, Manufacturing and Controls (CMC) developability of antibodies against a specific target are typically established for antibodies obtained from one platform only. In this study, monoclonal antibodies (mAbs) cross-reactive against human and cynomolgus LAMP1 were derived from the human immunoglobulin transgenic TRIANNI mouse and OmniChicken^®^ platforms and assessed for their specificity, sequence diversity, ability to bind to and internalize into tumor cells, expected immunogenicity and CMC developability. Our results show that the two platforms were complementary at providing a large diversity of mAbs with respect to epitope coverage and antibody sequence diversity. Furthermore, most antibodies originating from either platform exhibited good manufacturability characteristics.

## Introduction

Lysosome-associated membrane protein 1 (LAMP1) is a type I transmembrane protein composed of a large highly glycosylated luminal domain with 18 potential N-glycosylation sites and 6 O-linked oligosaccharides, a transmembrane domain, and a small cytoplasmic tail [1]. Given the resistance to various hydrolytic enzymes conferred by the complex carbohydrates and its abundance in lysosomal membranes, LAMP1 was—together with lysosome-associated membrane protein 2 (LAMP2)—initially considered to function as a barrier to protect lysosomal membranes from the lytic luminal environment [1, 2]. Furthermore, although LAMP1 is absent from the cell surface of most normal cells [3], it is expressed at the cell surface of activated cytotoxic lymphocytes and protects these cells from degranulation-associated suicide [4]. Finally, LAMP1 is expressed at the cell surface of tumor cells [5], and has been shown to play a role in cell-adhesion and tumor progression [6].

By immunizing mice with patient-derived xenograft (PDX) from a colon cancer patient and screening for antibodies specifically staining tumor plasma membrane, we have previously identified several anti-LAMP1 antibodies [7]. One of these, Ab1, bound to the luminal domain of human LAMP1 with nanomolar affinity. Subsequent immunohistochemistry using Ab1 demonstrated limited cell surface expression of LAMP1 in normal tissues while moderate to high membrane expression was found in a number of breast, colorectal, gastric, prostate, lung and ovary tumors. A humanized version of Ab1, humAb1, displayed rapid internalization into LAMP1-expressing HCT116 and colo205 cells. HumAb1 conjugated to DM4 maytansinoid derivative showed anti-tumor efficacy in pre-clinical studies when administered to mice bearing cell surface LAMP1 positive patient-derived tumors [7, 8].

Monoclonal antibodies represent the largest class of biopharmaceutical products [9, 10]. To minimize their immunogenicity in patients, mAbs intended for therapeutic applications should be as similar to native human antibodies as possible. Genetically engineered animals expressing a human immunoglobulin repertoire are a growing source of fully human therapeutic antibodies approved for human use [11]. While transgenic mouse strains have been used most frequently to date [12], the range of species that have been genetically modified and used for the generation of fully human antibodies has expanded to include rats, rabbits and cows [13], and most recently chickens [14]. Since chickens are phylogenetically separated from mammals, their proteins share less sequence homology with those of humans and human proteins are often immunogenic. Furthermore, human proteins may readily elicit antibodies in chicken that are cross-reactive with homologs in mammalian species, such as mice and cynomolgus monkeys, that are relevant to pre-clinical mechanism-of-action and toxicology studies. Finally, due to inherent differences between the immune systems of chickens and mammals, different antibody specificities may be obtained from the different platforms.

Next to ´humanness´ and specificity, developability is a key aspect of mAb therapeutics as their development entails challenges associated with CMC development such as aggregation, viscosity, susceptibility to chemical degradation and insufficient product stability. Such development risks are often associated with the intrinsic properties of the antibody candidates [15] and may vary depending on the transgenic platform used to generate the mAbs.

Here we report a comparative study of anti-LAMP1 mAbs generated in human immunoglobulin transgenic mouse (TRIANNI [16]) and chicken (OmniChicken^®^ [14]) platforms. Given the sequence identity varying from 48 to 74% between human and mouse LAMP1 subdomains and 32 to 61% between human and chicken LAMP1 subdomains, both platforms were considered suitable to generate antibodies to human LAMP1 that are anticipated to be cross-reactive with cynomolgus monkey LAMP1 which is 96% identical to human LAMP1. The OmniChicken strain used in this study expresses a single human immunoglobulin structural framework (V_H_3-23/Vκ3–15) consisting of VH and VL regions with diversity generated by gene conversion from inserted synthetic human pseudogenes. In the TRIANNI platform, mice produce human variable region antibody repertoire where the human variable genes are expressed from 44 V_H_ associated with D_H_ and J_H_, 39 Vκ associated with Jκ or 38 Vλ associated with Jλ. With the TRIANNI strain used in this study, the mice produce human V_H_ / human Vκ variable domain antibodies and human V_H_ / murine Vλ using V(D)J recombination [16].

Antibodies generated in each platform were screened for their binding to recombinant human and cynomolgus LAMP1 and 37 mAbs were assessed head-to-head for their reactivity with human and cynomolgus LAMP1 on different supports, their propensity to be internalized into LAMP1-expressing cells, and their human germinality. Based on their overall characteristics, 15 mAbs were further evaluated for their manufacturability and stability in a developability study.

## Results

### Generation of anti-LAMP1 antibodies

Antibodies against human LAMP1 were generated using the OmniChicken and TRIANNI transgenic platforms. OmniChickens were immunized with the extracellular domain of LAMP1 produced in HEK293 cells and single B cells from spleens were screened with the Gel Encapsulated Microenvironment (GEM) assay [14] using beads coated with either human or cynomolgus LAMP1 protein. One group of OmniChicken was immunized with human LAMP1 and the GEM assay was performed with cynomolgus LAMP1-coated beads. Another group was immunized with human and cynomolgus LAMP1 proteins and the GEM assay was performed with human LAMP1 and cynomolgus LAMP1-coated beads. A total of 36 unique immunoglobulin sequences were retrieved and formatted into scFv-Fc. Upon transient production in HEK293 cells, 35 of the 36 clones were found to bind both recombinant human and cynomolgus LAMP1 by ELISA. Based on binding, 24 clones were reformatted into human IgG and human/cynomolgus LAMP1 cross-reactivity was confirmed for 22 clones.

One group of TRIANNI mice was also immunized with the extracellular domain of human LAMP1 produced in HEK293 cells after which either hybridomas were generated and screened for reactivity with human and cynomolgus LAMP1 protein by ELISA, or single IgG-kappa B cells specific for cynomolgus LAMP1 were sorted by flow cytometry. Another group of mice was immunized with colorectal PDX and single IgG-kappa B cells specific for cynomolgus LAMP1 were sorted by flow cytometry. From both groups combined, a total of 20 unique sequences were retrieved by RT-PCR and reformatted into full-length human IgG. Following transient production in HEK293 cells, all clones bound to both human and cynomolgus LAMP1 as determined by ELISA. Based on sequence diversity, 19 OmniChicken-derived and 18 TRIANNI-derived anti-LAMP1 mAbs were selected for further characterization (S1 Fig).

### Affinity and cross-reactivity of anti-LAMP1 antibodies

**Fig 1:**
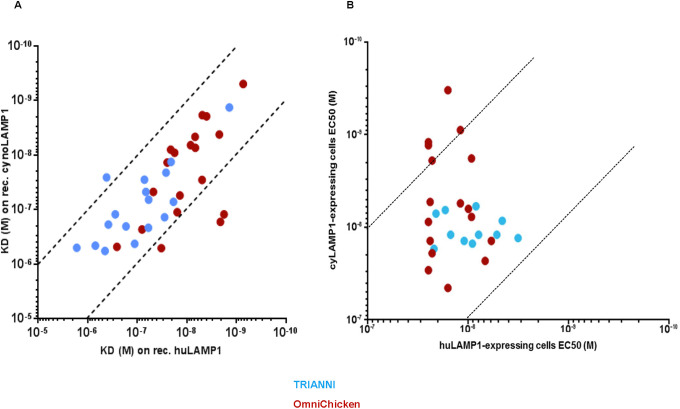
Majority of selected mAbs are cross-reactive with human and cynomolgus LAMP1. Binding of anti-LAMP1 antibodies to recombinant human and cynomolgus LAMP1 as measured by SPR (A) and to cell-surface expressed human and cynomolgus LAMP1 as measured by FACS (B). OmniChicken- and TRIANNI-derived mAbs are represented as brown and blue circles, respectively. Dotted lines indicate K_D_ ratios (human/cynomolgus) of 0.1 and 10.

Affinity of the 37 selected antibodies for recombinant human, cynomolgus and murine LAMP1 proteins produced in HEK293 cells was determined by surface plasmon resonance (SPR) and affinity for cell-surface expressed human and cynomolgus LAMP1 was assessed using flow cytometry. Consistent with the ELISA results, all antibodies bound to both human and cynomolgus LAMP1 (Fig 1A). One antibody, TRIANNI-485H, did not bind to recombinant LAMP1 used in SPR, but this antibody did bind to both human and cynomolgus cell-surface expressed LAMP1 used in flow cytometry. Interestingly, the mean affinity of the OmniChicken-derived mAbs for human LAMP1 (K_D_ 29 nM) was approximately 10-fold higher than that of the TRIANNI-derived mAbs (K_D_ 264 nM) (p < 0.0001) (S2 Fig panel A, top). However, although still significant, the difference in affinity for cynomolgus LAMP1 was much smaller (mean K_D_ 96 nM vs 183 nM, p = 0.0280) (S2 Fig panel A, bottom). Furthermore, the affinity for cell-surface expressed human LAMP1 as determined by flow cytometry was slightly higher for the TRIANNI-derived mAbs compared to the OmniChicken-derived mAbs (mean EC_50_ 11 nM vs 17 nM, p = 0.02116) while no difference was observed in the affinity for cell-surface expressed cynomolgus LAMP1 (S2 Fig panel B). Of note, while none of the TRIANNI-derived mAbs exhibited binding to murine LAMP1, 8 of the OmniChicken-derived mAbs bound the murine antigen with K_D_´s ranging from 2.5 nM to 685 nM. Furthermore, 2 of the OmniChicken-derived mAbs and 7 of the TRIANNI-derived mAbs bound neither to human nor cynomolgus LAMP1 when expressed at the cell-surface. Importantly, 32 of the 37 mAbs had a K_D_ ratio (human/cynomolgus) between 0.1 and 10, and the EC_50_ ratio (human/cynomolgus) was also within this range for 23 of the 28 mAbs that bound both human and cynomolgus cell-surface expressed LAMP1 (Fig 1A and 1B). Ratios within this range mean that differences in affinity can be compensated by adjusting the dose in cynomolgus monkey toxicity studies and the vast majority of the mAbs thus showed relevant degrees of human-cynomolgus cross-reactivity.

### Epitope diversity of anti-LAMP1 antibodies

**Fig 2:**
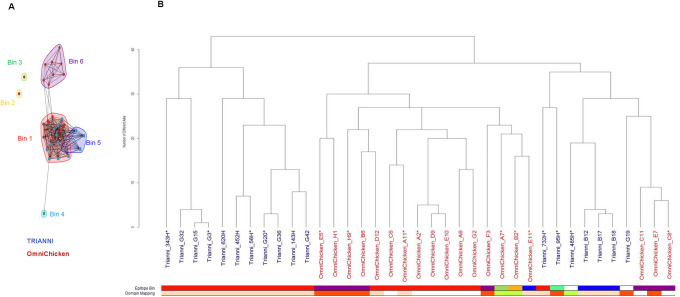
OmniChicken- and TRIANNI-derived mAbs target similar as well as distinct epitopes on LAMP1. (A) Epitope binning shows 6 epitope bins, two of which contain both OmniChicken- and TRIANNI-derived mAbs. Round-shaped spots indicate antibodies for which competition information was available regardless of the presentation (immobilized and in solution). Square-shaped spots indicate antibodies for which competition information was available in only one direction (mostly in solution). B) Paratope sequence tree of the 37 antibodies constructed using the Levenshtein distance based on the amino-acid differences within the six complementarity-determining regions (CDRs) defined according to international ImMunoGeneTics information system^®^ (IMGT). Bars at the bottom indicate the epitope bin to which each antibody is assigned with colors corresponding to those in panel A, and the LAMP1 domain to which each antibody binds with colors corresponding to those in Fig 3. Two antibodies, TRIANNI_485H and TRIANNI_G19, bound poorly in this experiment and were not assigned to an epitope bin. Asterisks (*) indicate mAbs further characterized in the developability study.

High throughput SPR (Carterra) was used to perform epitope binning experiments. Six epitope bins were identified (Fig 2A). One bin was populated with both OmniChicken- and TRIANNI-derived mAbs, whereas three bins were specific to OmniChicken-derived mAbs and two contained TRIANNI-derived mAbs. Analysis of the paratope sequences showed that each transgenic platform had yielded both closely related as well as diverse sequences and that the overall diversity was higher between platforms. As expected, clones with a few amino-acid sequence differences in their CDRs belonged to the same epitope bin (Fig 2B).

**Fig 3:**
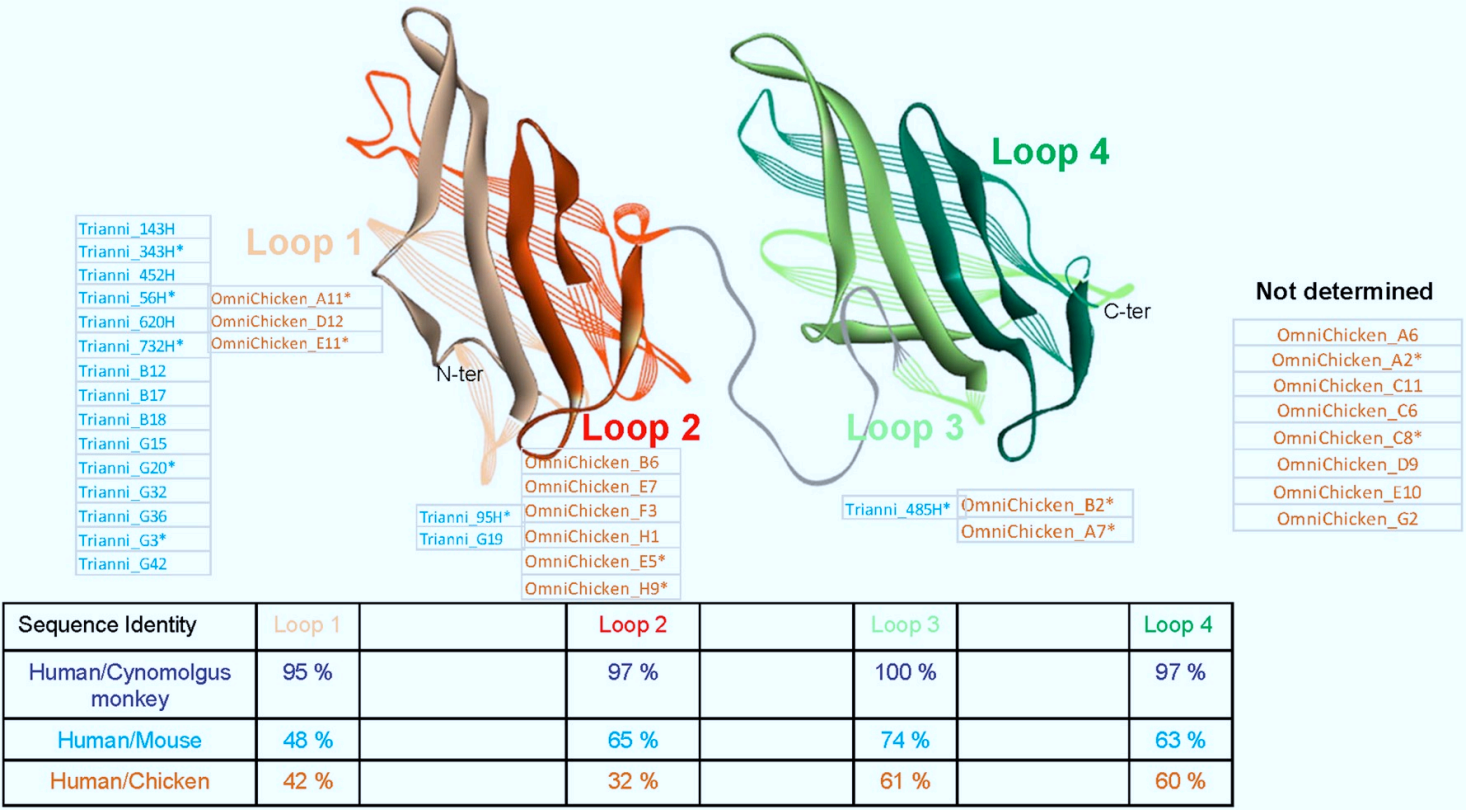
Domain mapping of anti-LAMP1 mAbs. Structure model of the extracellular domain of human LAMP1 with Loop 1 in light brown, Loop 2 in darker brown, Loop 3 in green and Loop 4 in darker green. The names of the anti-LAMP1 mAbs binding to each of the loops are indicted. Table shows the sequence identity to human LAMP1 for each of the four subdomains (loops) of the cynomolgus, murine and chicken LAMP1 homologues. Asterisks (*) indicate mAbs further characterized in the developability study.

Crystal structures have shown that the extracellular domain of human LAMP1 consists of two beta-prism shaped subdomains separated by a hinge [17]. The presence of two disulfide bonds in each of these subdomains permits a further definition of four loops. In order to map to which domain each of the anti-LAMP1 mAbs bound, we constructed four chimeric LAMP1 proteins by replacing one luminal domain or loop subdomain of human LAMP1 by its murine counterpart. Human, mouse and the chimeric LAMP1 proteins were produced in HEK293 cells and antibody binding was assessed by ELISA (Fig 3). Most TRIANNI-derived mAbs (15 out of 18) bound to the first loop of the first luminal domain (Loop 1), whereas 2 mAbs bound to the second loop of the first luminal domain (Loop 2) and one mAb bound to the first loop of the second luminal domain (Loop 3). No antibodies were found to be directed to the second loop of the second luminal domain (Loop 4). With respect to Loops 1 to 3, an expected inverse correlation was observed between the sequence identity between human and murine LAMP1 and the number of antibodies found. The results for the OmniChicken-derived mAbs suggest a similar trend with most of the mapped antibodies binding to Loop 2 which shares the lowest sequence identity with chicken LAMP1 (Fig 3). However, the binding domain of 8 of the 19 OmniChicken-derived mAbs could not be determined because they appeared to be cross-reactive with murine LAMP1.

Next, the binding of the anti-LAMP1 mAbs to colo205 and LAMP1-expressing HCT116 tumor cell lines and to three PDX derived from lung or bladder was tested by flow cytometry. More than half of the antibodies from each platform bound to all of the tested cells, with 83% of the TRIANNI-derived and 68% of the OmniChicken-derived mAbs binding to all three different PDXs that were tested (S3 Fig). The percentage of OmniChicken-derived mAbs that bound to LAMP1-expressing HCT116 cell line was slightly higher than that of the TRIANNI-derived mAbs (84% vs 72%). Since tumor cell lines and PDXs express LAMP1 with different glycan composition, making certain epitopes more or less accessible, these results suggest that different epitopes are accessible on tumor cell lines and PDXs for mAbs derived from the 2 platforms.

### Internalization of anti-LAMP1 mAbs

**Fig 4:**
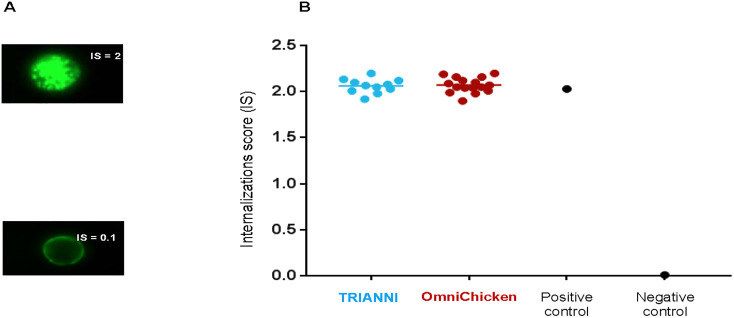
Anti-LAMP1 antibodies are readily internalized by LAMP1-expressing HCT116 cells. (A) Representative images of tumor cells that internalized antibodies with high (IS = 2), and no internalization (IS = 0.1). Images shown are from antibodies unrelated to this study. (B) Internalization scores of anti-LAMP1 mAbs derived from the TRIANNI (blue circles) and OmniChicken (brown circles) platforms and positive and negative controls on LAMP1-expressing HCT116 cells. Horizontal lines indicate means.

We next assessed the rate at which the mAbs that bound to LAMP1-expressing HTC116 cells were internalized. Using a fluorescently labeled anti-human IgG and imaging flow cytometer, the internalization rate was determined as a function of the fluorescence intensity inside and around individual cells and expressed as an internalization score (IS) calculated on 5 000 individual cells (for details see Materials and methods). In previous work [7] we have established that a high rate of internalization of antibodies into LAMP1-expressing tumor cells such as Colo205 cell line corresponds to an IS of around 2. All the antibodies were internalized by LAMP1-expressing HCT116 cells with internalization scores close to this value, the IS score is 2.05 **±** 0.08 for TRIANNI-derived anti-LAMP1 and 2.07 **±** 0.08 for OmniChicken-derived anti-LAMP1. It shows showing that all mAbs had a very good propensity for internalization (Fig 4).

### Sequence analyses and immunogenic potential of anti-LAMP1 mAbs

**Fig 5:**
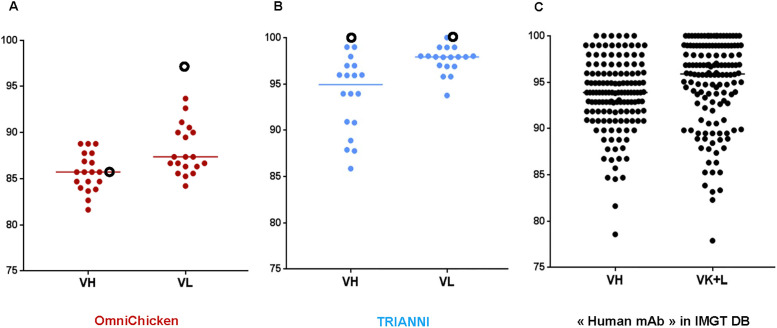
IGV protein identity to human germline of OmniChicken- and TRIANNI-derived anti-LAMP1 mAbs compared to the IGV protein identity of a panel of 140 human antibodies tested for therapeutic applications in humans. Shown are the identities for the heavy and light chain V gene sequences of 19 OmniChicken-derived mAbs (brown), 18 TRIANNI-derived mAbs (blue) and 140 human antibodies tested in the clinic (green). Black empty circles represent the SynVH-C and SynVκ-C sequences inserted in the OmniChicken platform and the 44 V_H_ and 39 V_L_ sequences inserted in the TRIANNI platform.

The germinality index of the 37 anti-LAMP1 mAbs, as well as of 140 therapeutic mAbs tested in humans extracted from the IMGT database for therapeutic monoclonal antibodies (www.imgt.org/mAb-DB/query) [18], was calculated as the percentage of identity to the closest functional human germline of the complete V gene. Hereto, the analysis included the amino-acid sequence of the variable domains of the heavy (V_H_) and light chain (Vκ) from the first framework to the beginning of the CDR3. The inserted sequences in the TRIANNI mouse were 100% identical to the human germlines while the generated anti-LAMP1 antibodies presented a decrease in germinality index as expected from V(D)J recombination and somatic hypermutations (Fig 5). In contrast, the V_H_ sequence inserted in the SynVH-C OmniChicken strain was derived from a human B cell library and is 86% identical to the closest human germline gene, V_H_3-23, and the median identity of the V_H_s of anti-LAMP1 mAbs was similar. This result was expected considering the 97% human identity of the synthetic human pseudogenes inserted into this OmniChicken strain and the prevalent role of gene conversion in chicken [19]. No sequence donated by chicken pseudogenes was observed. As shown in Fig 5, the Immunoglobulin G V (IGV) protein identity to human germlines was the lowest for the OmniChicken-derived mAbs with median identities of 86% and 87% for V_H_ and Vκ, respectively, compared to 95% and 98% for the TRIANNI-derived mAbs, and 94% and 96% for the 140 mAbs from clinical trials. However, the percentages of identity of the variable domains of the OmniChicken-derived anti-LAMP1 mAbs still fall within the range of those observed among the 140 therapeutic antibodies.

**Fig 6:**
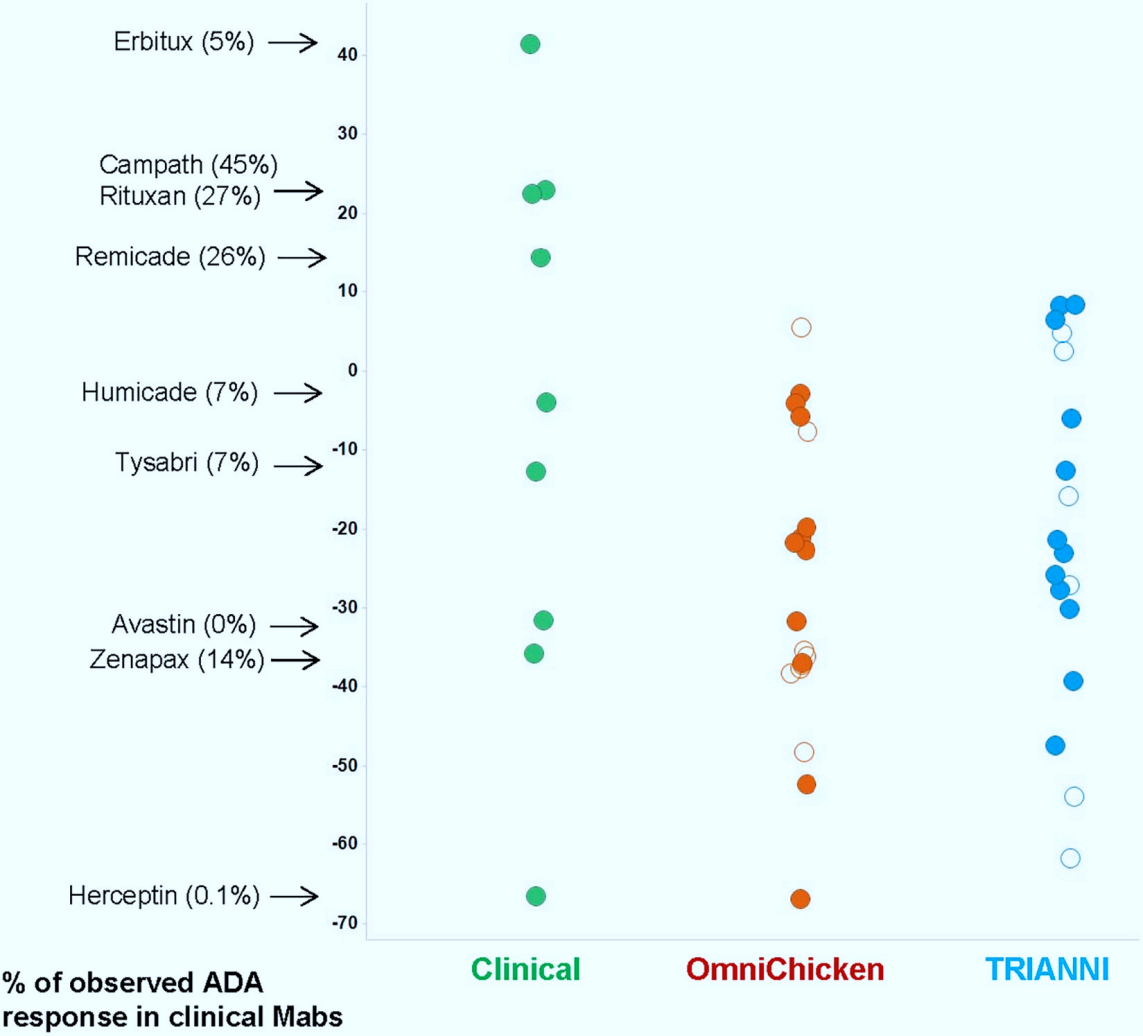
EPIVAX scores for the OmniChicken- (brown), TRIANNI-derived (blue) and a panel of antibodies that have been tested in humans (green). For this latter group the reported % of ADA observed in clinical trials is indicated. Empty circles represent the mAbs selected for the developability study.

The immunogenic potential of the 37 mAbs was subsequently assessed using the Epivax tool [20, 21]. The EpiMatrix adjusted scores were calculated for the 37 mAbs as well as for a panel of antibodies that have been tested in humans and for which anti-drug antibodies (ADA) responses have been reported (Fig 6). The results for the ´clinical´ mAbs illustrate the trend that low EpiMatrix scores are associated with low ADA responses. Since the scores for the large majority anti-LAMP1 mAbs are relatively low, the ADA responses to these antibodies may be expected to be low.

Finally, the amino-acid sequences of the anti-LAMP1 mAbs were checked for the presence of problematic attributes (e.g. post translational modifications in CDRs) associated with known manufacturability, efficacy or safety concerns. A few potential asparagine deamidation, aspartic acid isomerization and methionine/ tryptophan oxidation sites were predicted while no unpaired cysteine residues or N-glycosylation sites were found within the variable domains.

### Evaluation of developability

Fifteen of the 37 anti-LAMP1 mAbs were further characterized with respect to their manufacturability and stability. Antibodies were selected to provide a good representation of the sequence diversity, epitope bins and subdomain specificity of the entire panel. Selected mAbs are Omnichicken_A2 _A7, _A11, _B2, _C8, _E5, _E-11, _H9 clones and TRIANNI_56H, _95H, _343H, _485H, _732H, _G3, _G20 clones as indicated in Figs 2, 3 and 6. All selected mAbs exhibited good production yields averaging >100 mg/L following transient expression in HEK293 cells (Table 1). After purification by protein A affinity and size exclusion chromatography (SEC), purity and integrity were evaluated by SEC and Mass Spectrometry (MS), respectively. All purified mAbs were within developability specifications. Binding was also confirmed by SPR for 14/15 mAbs and by FACS for the one TRIANNI- derived antibody (TRIANNI_485H) that did not bind to recombinant LAMP1.

**Table 1:** Developability of OmniChicken- and TRIANNI-derived anti-LAMP1 mAbs.

	Parameter	Assay	Criterium	OmniChicken	TRIANNI
Characterization	Yield	Protein A		132 ±16 mg/L	106 ± 23 mg/L
	Binding	SPR	Binding	8/8	6/7^a^
	Purity	SEC	HMWs + LMWs < 5%	8/8	7/7
	Thermal stability	NanoDSF	Tonset > 60°C	7/8^b^	7/7
			Fab Tm > 75°C	8/8	5/7^c^
	Integrity	MS	Expected value	8/8	7/7
Physical stability (14 days at 40°C)	Integrity	MS	Expected value	8/8	7/7
	Binding	SPR	Confirmation of binding	8/8	6/6
	Mass profile	SEC	ΔT0 < 10%	8/8	6/7^d^
Low pH stability (4 hours at pH 3.7)	Mass profile	SEC	< 1% increase HMWs	7/8^e^	7/7

^a^ TRIANNI_485H: No binding by SPR, but binding to cell-expressed LAMP1

^b^ OmniChicken_C8: Tonset 56°C

^c^ TRIANNI_56H, TRIANNI_G20: FabTm not determined

^d^ TRIANNI_95H: 19% HMWs + LMWs

^e^ OmniChicken_H9: 5% increase in HMWs after 4 hours at pH 3.7

Since thermostability is an intrinsic property of mAbs that can influence product stability during manufacturing and storage [15], the midpoint temperature of unfolding of the mAb (Tonset) and of the Fab domain (Fab Tm) were analyzed. Thermostability was assessed just after purification by nano-DSF (Differential Scanning Fluorimetry). All mAbs passed the Tonset criterion (> 60°C) except one OmniChicken-derived mAb, whereas all but two TRIANNI-derived mAbs passed the Fab Tm criterion (> 75°C) (Table 1). Physical stability was analyzed by assessing integrity, binding and mass profile after incubation at 40°C for 14 days. Except for one TRIANNI-derived mAb that presented a significant differential increase of high and low molecular weight species (HMW and LMW) following incubation at 40°C, all antibodies passed the quality criteria (Table 1). The production process of therapeutic antibodies usually includes incubation at low pH in order to inactivate potentially present viruses [15]. Therefore, we assessed the effect of incubating the mAbs at pH 3.7 for 4 hours on their stability. SEC analysis indicated that 14/15 mAbs passed the criterion of < 1% raise in HMW (Table 1). Overall very few mAbs were out-of-specifications, and no correlation was found with potentially problematic residues in their sequences as such residues were also present in mAbs that fulfilled all developability criteria. Clones within specifications are Omnichicken_A2, _A7, _A11, _B2, _E5, _E-11 and TRIANNI_56H, _343H, _732H, _G3, _G20. These results show that the majority of the antibodies from both platforms had good manufacturability characteristics.

## Discussion

Several transgenic animal platforms are now well established to generate human antibody therapeutics [13].

Mouse, rat, and chicken transgenic platforms were all shown to be able to deliver high affinity antibodies against a variety of targets [11–13, 22, 23]. However, no head-to-head comparison between transgenic platforms had yet been reported. In this study, we compared two recently developed transgenic animal platforms with respect to antibody affinity, multi-species cross reactivity, epitope coverage and developability/manufacturability for a subset of monoclonal antibodies.

We have identified human cynomolgus cross-reactive anti-LAMP1 mAbs with affinities in the nM range from both the OmniChicken and TRIANNI transgenic platforms. While some antibodies from both platforms grouped into the same epitope bins and thus target the same or spatially proximal epitopes, epitope bins specific for antibodies from each platform were also identified, meaning that the platforms were complementary with respect to antibody specificity.

Interestingly, 8 mAbs derived from the OmniChicken platform were found to bind—next to human and cynomolgus LAMP1—also to mouse LAMP1, despite the fact that the immunization strategy and screening were not designed to identify antibodies cross-reactive to mouse LAMP1. Since different mammal species are often used for pre-clinical mechanism-of-action and toxicology studies, such widely cross-reactive antibodies are particularly attractive, and the ability of the OmniChicken platform to yield such antibodies illustrates the advantage of its phylogenetic distance to mammals. The advantage of this phylogenetic distance can be expected to be even larger for targets that are more conserved among mammals as such targets will be less immunogenic in those species.

Most of the TRIANNI-derived antibodies bind to the first loop of the first luminal domain, in accordance with the fact that this region of the LAMP1 protein is least conserved between humans and mice. Although 8 of the OmniChicken-derived mAbs could not be mapped due to their cross-reactivity with murine LAMP1, the majority of the ones that were mapped bind to Loop 2, which is the region of LAMP1 that shares the lowest sequence identity between humans and chicken.

We observed a good correlation between the epitope binning and domain mapping experiments as all the mAbs targeting Loop 1 are in epitope bins 1 and 5 while Loop 2-binding antibodies are all, with one exception (TRIANNI_95H which is in a bin on its own), in bin 6 (Fig 2). None of the antibodies binding Loop 3 are in any of the previously mentioned bins, although that of TRIANNI_485H could not be determined due to its lack of binding to recombinant LAMP1. Considering the observed correlation between the epitope binning and domain mapping experiments, and the paratope sequence relationships, we can speculate to which domains the 8 OmniChicken-derived mAbs that could not be mapped using the human-mouse chimeric LAMP1 proteins are likely to bind: Six of these antibodies share high sequence identity with, and are in the same epitope bin (e.g. bin 1) as, antibodies that bind to Loop 1 and are thus likely to bind this domain (Fig 2 and S4 Fig). The other 2 mAbs are closely related to, and share their epitope bin (e.g. bin 6) with an antibody that binds to Loop 2 and presumably thus bind this domain (Fig 2 and S4 Fig). Using a similar logic, we can speculate that mAb TRIANNI_G19 which bound too poorly in the binning experiment to assign it to an epitope bin, is likely to belong to bin 6, as it was shown to bind to Loop 2 and shares a high sequence similarity with other antibodies in this epitope bin. No antibodies directed against Loop 4 were identified in this study, which is in line with previous work in our lab suggesting a poor immunogenicity of this loop despite relatively poor sequence conservation between the considered species.

There was large sequence diversity among the antibodies of each platform and this diversity was obtained by different mechanisms. OminiChicken platform led to mAbs with more than 30 different amino-acids in 6 IMGT CDR paratopes. Although only one pair of VH/Vk genes was introduced in the transgenic chicken, diversity was generated by gene conversion and somatic mutations [19]. With the TRIANNI platform, mAbs with more than 30 different amino-acids in 6 IMGT CDR paratopes were also obtained via VDJ recombination and somatic mutations from 5 VH and 7 VL human germlines. Although the human germinality index was greater for mAbs derived from TRIANNI mice than for those derived from the OmniChicken platform, OmniChicken antibodies were still within the range of human therapeutic mAbs that have been assessed in the clinic.

All of the antibodies that could be tested in our internalization assay showed a high propensity for internalization and therefore potential promise for therapeutic application. Since development risks are often associated with intrinsic properties of drug candidates, we subjected a selection of the antibodies to a developability study. Criteria were reached for the production and thermal and pH stability for the majority of antibodies from each platform and therefore a positive outcome for manufacturability can be anticipated.

In conclusion, we have shown that by using both the OmniChicken and TRIANNI platforms to generate antibodies to human LAMP1, we obtained a panel of mAbs exhibiting favorable developability traits with a broader range of epitope specificity and sequence diversity than we would have obtained from either platform alone.

## Materials and methods

### Immunization of transgenic animals

The OmniChickens used in this study express pre-rearranged human VH3-23/JH4 and VK3-15/JK4 variable regions spliced to chicken constant regions, with chicken non-coding sequences such as promoters and introns. Immunizations were performed as previously described [14] at Ligand (Emeryville, CA, USA) in accordance to Institutional Animal Care and Use Committee (IACUC)-approved protocols and under supervision of the IACUC.

Immunizations of the TRIANNI (San Francisco, CA, USA) strain [16] were performed at Sanofi (Vitry-sur-Seine, France) and overseen by a licensed veterinarian. Institutional Animal Care and Use approval was obtained by the CEPAL committee #PEA 2012–0077. The TRIANNI is a C57BL/6 strain that transgenically expresses a complete repertoire of fully human immunoglobulin gamma (IgG) and immunoglobulin kappa (IgK) V(D)J genes, but retain mouse regulatory genomic sequences [16]. Immunizations were performed using the classical method as described by Wennerberg A.E *et al*., [24]. Six-eight weeks old female mice each received three rounds of intraperitoneally injections of purified LAMP1 over a course of 41 days at intervals of 3–4 weeks. Hundred μg of human LAMP1 protein emulsified in Titermax’s adjuvant (TierMax Gold Adjuvant; Sigma T2684) was administered intraperitoneally. Three days after the last injection, mice were sacrificed and spleens were isolated for antibody generation. One group of mice was immunized with patient-derived xenograft CR-IGR-034P using classical protocol described above using 10 million cells per round. Alternatively, the RIMMS method as described by Kilpatrick *et al*., [25] was used. In this approach, 6–8 weeks old female mice each received four rounds of subcutaneous injections of purified LAMP1 over a course of 14 days at intervals of 3–4 days. Ten μg of human LAMP1 emulsified in Titermax’s adjuvant (TierMax Gold Adjuvant; Sigma #T2684) were administered subcutaneously to six sites proximal to draining lymph nodes, along the back of the mice and to six juxtaposed sites along abdomen. Four days after the last injection, mice were sacrificed. Bilateral popliteal, superficial inguinal, axillary and branchial lymph nodes were isolated for antibody generation.

### Cloning of antibody sequences from hybridoma or single B cells

Following mice sacrifice, B cells were isolated from spleen or lymph nodes according to classical or RIMMS procedures, respectively. Single-cell suspension was fused with P3X63-AG8.653 myeloma cells using the polyethylene glycol fusion method [26]. After incubation at 37° C for 16–24 hours, the resulting cell preparation was transferred into selective semi-solid medium, plated out into Petri plates and incubated at 37°C. Ten days after initiation of selection, isolated colonies were picked, amplified using ClonePix^™^ 2 Mammalian Colony Picker. Paired VH/VL genes were retrieved from 100 clonal cells by RT-PCR and sequenced with a similar protocol as for single-B-cell below.

In a second method, antigen-specific IgG+ memory B cells were sorted as described by Starkie *et al*., [27] After mice sacrifice, B cells were enriched from splenocytes using Pan B Cell Isolation Kit II (Miltenyi; 130-047-301) according to manufacturers’ instructions. After enrichment, cells were suspended in buffer solution and stained with 1 μg per 10^8^ cells of the following antibodies (BioLegend): Rat anti-mouse IgG brilliant violet 421, rat anti-mouse CD45R brilliant violet 785, rat anti-mouse Ig light chain κ PE and rat anti-mouse IgD and rat anti-mouse IgM brilliant violet 605 (dump channel). Dual human LAMP1::Halotag-histag and cyno LAMP1::Halotag-histag proteins were detected using ligand-Halotag-FITC and ligand-Halotag-APC, respectively. Cells were separated using BD FACSAria^™^ Fusion for flow cytometric cell sorting to isolate human/monkey cross reactive IgG-LAMP1 specific B cells. Single cells were directly sorted into PCR tubes to amplify by RT-PCR and sequence paired VH / VL genes using a protocol similar to Tiller *et al*., [28].

For the OmniChicken, antigen specific titers were assessed on a bi-weekly basis by ELISA. Following sacrifice, spleens were taken and cryopreserved in 10% FBS-RPMI. For each GEM screen, 1–2 x 10^7^ cells were thawed and incubated at 37 °C with cyno or human LAMP-coated polystyrene beads and anti-chicken IgY (H+L)-AF 594. Following incubation, antigen positive GEMS were selected, lysed and single cell RT-PCR performed as previously described [14, 23]. Natively paired VH/VK were then cloned in a scFvFc format and expressed in EXPI293 cells. Antigen specificity was confirmed by ELISA, and a select group of clones was reformatted to full IgG, then reconfirmed by ELISA.

### Protein and mAb production

Nucleic acid sequences coding for LAMP1 extracellular domains fused to his-tag or Halotag-histag at the C-terminal or coding for the antibody heavy or light chains were cloned into mammalian expression plasmids under the CMV enhancer/promoter and the SV40 polyA signal. Resulting plasmids were transfected into HEK293 cells (Thermo Fisher Scientific; K9000-10) using FreeStyle^™^ MAX 293 Expression System according to the manufacturer’s instructions. LAMP1 proteins were purified by immobilized metal affinity chromatography (Chelating Sepharose, 17-0575-01 GE Healthcare) and stored in PBS after concentration and buffer exchange (Sephadex G-25 column, GE Healthcare).

For initial characterization, mAbs were produced at 30 mL scale, purified by protein A affinity chromatography and stored in PBS after desalting on mini trap Sephadex G-25 column. For the developability study, mAbs were produced at 1 L scale and purified by a two-step process including protein A affinity chromatography (HiScreen MabSelect Sure protein A, GE Healthcare, 28-9269-77) and size-exclusion chromatography (HiLoad 26/600 superdex 200 pg, GE Healthcare, 28-9893-36).

### PDX expressing LAMP1

A large collection of Patient-derived colorectal, lung and bladder cancer tumors was collected, after patient’s informed consent, in French medical centers. The tumor samples were engrafted on Swiss nude mice; tumors reaching a volume of 800 to 1500 mm3 were frozen prior to characterization and use in this study [29].

### SPR

Binding kinetics of mAbs were measured by SPR using a Bruker SPR-32 instrument. An anti-human Fc antibody (Human Antibody Capture Kit, GE LifeSciences) was covalently coupled on HCA sensorchip (Bruker). Spots were first activated by a 7-min pulse of 1:1 400 mM EDC:100 mM NHS mixture (Amine Coupling Kit, GE LifeSciences). The anti-human Fc antibody was diluted to 20 μg/mL in 10 mM acetate pH 5.0 and coupled for 7 min. Uncoupled sites were then deactivated by a 7-min pulse of 1 M ethanolamine pH 8.5. 6000–8000 RU of antibodies were typically observed on all spots. Following surface preparation, antibodies were diluted to 2 μg/mL and captured for 1 minute at 10 μL/min up to ≈ 300 RU. Serial concentrations of human, cynomolgus and murine LAMP1 protein (3.7–300 nM) were then injected for 5 minutes at 30 μL/min. Dissociation was monitored for 15 min. Surfaces were regenerated by a 30 s pulse of 3 M MgCl2. Antibodies and antigens were diluted in running buffer (HBS-EP+, 0.01 M HEPES pH 7.4, 0.15 M NaCl, 3 mM EDTA, 0.005% v/v Surfactant P20; GE LifeSciences). Sensorgrams were double-referenced by subtracting a reference spot and a buffer injection. They were then fitted using a 1:1 model with the MASS-2 Analyze software (Bruker). Statistical tests were performed with Prism software (GraphPad). An unpaired, two-tailed Mann–Whitney U test was used to analyze the differences between the groups. A p-value of < 0.05 was considered statistically significant.

### Epitope binning

Epitope binning experiments were performed using the LSA instrument (Carterra). mAbs were covalently coupled to a CMD50M chip (Carterra). Spots were first activated by a 10-min pulse of EDC/NHS mixture using the 96-channel printhead. mAbs were diluted to 10 μg/mL in 10 mM acetate pH 5.0 and coupled for 7 min using the 96-channel printhead. Uncoupled sites were then deactivated by a 10-min pulse of 1 M ethanolamine pH 8.5 using the single flowcell. Following surface preparation, a double injection composed of 100 nM human LAMP1 immediately followed by 100 nM anti-LAMP1 mAb was realized. After regeneration of the surface with two 15 s pulse of 10 mM glycine pH 2.0, another double injection of huLAMP1/mAb was realized. All samples were diluted in HBS-EP+ running buffer. The sensorgrams are analyzed using the Epitope Tool (Carterra). Antigen binding level is first normalized; antibody binding to antigen is then measured and normalized to build network plots.

### ELISA

Enzyme-linked immunosorbent assay (ELISA) plates (Nunc MaxiSorp) were coated with 5 μg/mL of LAMP1 in PBS overnight at 4°C. Plates were then washed 3 times with PBS containing 0.05% Tween-20 (PBS-T), and subsequently blocked with PBS-T plus 1% bovine serum albumin (BSA), for 20 min at room temperature. One hundred microliters of antibody at 5 μg/ml in PBS-T were added to the plates and incubated for 1 hr at 37°C. Afterwards, plates were washed as before, and binding of antibodies was detected by incubation of 100 μL of a 1:200 000 dilution of horseradish peroxidase-conjugated goat anti-human IgG antibody (Jackson ImmunoResearch; 109-035-098, West Grove, Pa.). Plates were washed with PBS-T five times. Antibody binding was visualized by adding TMB-H2O2 buffer and read at a wavelength of 450 nm using microplate reader Infinite F500 (Tecan).

### Flow cytometry

Recombinant murine preB 300.19 cell lines expressing human LAMP1 (clone 6645-hLAMP1-cl6) or cynomolgus monkey LAMP1 (clone 6646-cynoLAMP1-cl71) at the cell surface were used to measure the binding of anti-LAMP1 antibodies by flow cytometry. Cells were coated at 100,000 cells/well on 96-well plate (Falcon; 353910)) and 100 μL/well of antibody was added in 2-fold serial dilutions starting at 300 μg/mL up to 12 dilutions in assay diluent for 20 min at 4°C and washed two times with PBS 1% BSA. Binding of the antibodies was detected by 100 μL/well of Alexa Fluor^®^ 488 conjugated goat anti-human IgG (Jackson Immunoresearch; #109-545-098, West Grove, Pa.) for 15 min at 4°C and then washed two times with PBS 1% BSA. The antibody binding was evaluated after centrifugation and resuspension of cells by adding 150 μl/well PBS 1% BSA and read using Guava^®^ easyCyte 8HT Flow Cytometry 5 System. EC50 values were determined using BIOST@TSPEED software. Statistical tests were performed with Prism software (GraphPad). An unpaired, two-tailed Mann–Whitney U test was used to analyze the differences between the groups. A p-value of < 0.05 was considered statistically significant. Binding of anti-LAMP1 antibodies to two tumor cell lines HCT116-huLAMP1 and colo 205, and to three PDX PDX LUN NIC70, PDX LUN NIC025 and PDX SA BLA0025 was also determined by flow cytometry using the same protocol at the highest antibody concentration.

### Internalization score

An ImageStreamX Mark II multispectral imaging flow cytometer (Luminex Corp.) was used to determine the internalization score (IS) of anti-LAMP1 antibodies following binding to LAMP1-expressing colon HCT116 tumor cell line. Viable HCT116 cells (4×10^4^ cells) were seeded into wells of 6-well plates and incubated with 5 μg/mL of mAbs for 18 hours at 37°C/5% CO2. Cells were washed by centrifugation with PBS 1% BSA at 400 g for 3 minutes. Cells were fixed and permeabilized using 500 μL of BD Cytofix/Cytoperm buffer (BD Biosciences; 554722) on ice for 20 minutes. Cells were washed by centrifugation with 4 mL of Perm/Wash Cell buffer (BD Biosciences; 554723) at 400 g for 3 minutes. To test whether antibodies internalized, 1 mL of a 1:400 dilution of AlexaFluor488 conjugated goat anti-human IgG (Jackson Immunoresearch; 115-545-164, West Grove, Pa.) was incubated on ice for 20 minutes. After incubation, 4 mL of PBS 1% BSA buffer was added to wash, before centrifuging (400 g, 3 min). The supernatant was flicked from the plate before the cells were fixed with 150 μL 1% formaldehyde on ice for 20 minutes. The fluorescence of cells was analyzed with the ImageStream multispectral imaging flow cytometer using the Internalization feature. Five thousand events were acquired for each experimental condition and the corresponding images were analyzed using the IDEAS 5 image-analysis software as illustrated on S5 Fig.

The Internalization Score (IS) is then computed as follows:

$$IS=log\left(\frac{a}{1-a}\right),$$

where

$$a=\frac{m_{I}}{m_{I}+m_{B}}\frac{p_{I}}{P_{B}}$$

(B) = External and (I) = internal part of the cells

mI = Mean intensity of upper quartile pixels in I, mB = Mean intensity of upper quartile pixels in B

pI = Peak intensity of upper quartile pixels in I, pB = Peak intensity of upper quartile pixels in B.

### Antibodies protein sequence identity to human germline

To assign the closest human germline, a BLASTp was done against, IMGT/Gene database on functional alleles [30]. The percentage of identity to the human germline is calculated on the complete V gene. Human mAbs tested in clinics were retrieved from IMGT [18]. A paratope sequence tree was constructed using the Levenshtein distance using the statistical tool R. The Levenshtein distance is a string metric classically used for measuring the difference between two sequences and in this case within the six CDRs. It is calculated as the minimum number of single-character edits either insertion, deletion or substitutions required to change one character into another.

### V-domain T cell epitope In-silico prediction

The immunogenic potential of the antibodies was assessed using the Epivax tool. Briefly, variable domain (VH plus VL) protein sequences of antibodies analyzed by EpiMatrix were parsed into 9-mers and scored for predicted binding affinity against a panel of eight common Class II HLA alleles [21]. This score was adjusted for the presence of Tregitopes supposed to be tolerated or to prevent immunogenicity [20], and compared to a panel of therapeutic antibodies tested in humans for which observed ADA responses have been reported.

### High temperature and low pH stability

For the temperature stress experiment, mAbs concentration was around 5 mg/mL in 10 mM histidine pH 6. Storage at 40°C for 14 days, was performed in 1.5 mL vials incubated in ThermoMixer F1.5 (Eppendorf). For the low pH assessment, mAbs at around 5 mg/mL and pH 3.7 were obtained after dialysis in acetic acid and stored for 4 hours under smooth stirring at room temperature.

### Size-exclusion chromatography

Analytical SEC-HPLC was performed with a HPLC system (1260 HPLC system with PDA Agilent Technologies) and a 3-mL S200 column (Increase GL 5 x 150 mm; GE Healthcare 28-9909-45). Buffer for elution was 1X D-PBS (from 10-fold dilution of 10X D-PBS, Gibco 14200–067). The flow rate was set at 0.3 mL/min for 20 minutes at a column temperature of 24 °C. After buffer exchange into D-PBS, 30 μL of mAb were injected on the column and detection was recorded at 280 nm. Data were processed with ChemStation software (Agilent Technologies).

### Differential scanning fluorimetry

The Tonset and Fab Tm thermal stability parameters were determined in the same experiment using a Prometheus NT-48 instrument (NanoTemper Technologies). Standard nanoDSF NT capillaries were filled with 10 μL of protein solutions that were subjected to a linear temperature gradient from 20° to 110°C with a 1°C heating rate. For kinetic analysis of the thermal stability, various heating rate data sets were collected (0.1°, 0.5°, 1°, 2°, 5°C) and processed by AKTS thermokinetics software package. The experiments were repeated 3 times and the provided data are the averaged values with error bar being less than 0.05°C.

### Mass spectrometry

mAb samples were diluted to 1 mg/mL in 1X D-PBS or 10 mM histidine pH6.5 buffer before analysis. Mass of the entire mAb was determined as follows. Reversed-phase high performance liquid chromatography (RP-HPLC) was performed on a Waters Acquity UPLC system. The mobile phases consisted of water with 0.031% trifluoroacetic acid as solvent A and acetonitrile with 0.03% trifluoroacetic acid as solvent B. A Jupiter C4 column (2 x 150 mm, 5 μm particle size, 300 Å pore, Phenomenex), was used for the RP-HPLC time-of-flight (TOF) mass spectrometric (MS) analysis. The column eluent was directed in-line to a TOF mass spectrometer (Q-TOF Premier, Waters). The initial mobile phase was 20% solvent B, and then it was applied a gradient of 2.5% solvent B per min from 20–50% solvent B. The separation was performed at room temperature at a flow rate of 0.35 mL/min. The electrospray ionization mass spectra were analyzed using OpenLynx protein deconvolution software (Waters).

## Supporting information

S1 Fig Paratope sequence tree as in Fig 2 with the origin—method of immunization (classical (I, III) or RIMMS (II) for TRIANNI-derived mAbs), antigen (recombinant human LAMP1 (I, II) or PDX (III) for TRIANNI-derived mAbs and only recombinant human- (1), or both human and cynomolgus LAMP1 (2) for OmniChicken-derived mAbs) and selection (hybridoma (I) or single B cell sorting (II, III) for TRIANNI-derived mAbs, and GEM assay with only beads coated with cynomolgus LAMP1 (1) or with both beads coated with human- and beads coated with cynomolgus LAMP1 (2) for OmniChicken-derived mAbs)—of each of the 37 antibodies indicated.
(TIFF)

S2 Fig Affinities of the selected TRIANNI- and OmniChicken-derived antibodies for human and cynomolgus LAMP1 as determined by SPR (A) and FACS (B). Horizontal lines indicate means ± SEM.
(TIFF)

S3 Fig Accessible LAMP1 epitopes on tumor cells and PDXs. Percentage of TRIANNI- and OmniChicken-derived anti-LAMP1 mAbs binding to the indicated tumor cell line or PDX as determined by flow cytometry.
(TIFF)

S4 Fig Network plots for anti-LAMP1 antibodies showing the 6 established epitope bins as in Fig 2A, highlighting the antibodies that are cross reactive with murine LAMP1 (panel A) and the domain specificity of the antibodies (panel B) as in Fig 3. (TIFF)

S5 Fig Internalization score measurement. Representative data with labeled TRIANNI _G3 anti-LAMP1 onto LAMP1- expressing HCT116 cells. Panel A: Gating for high Max Pixel and Intensity for at least 5 000 labeled cells. Panel B: Data acquisition on the gated cells for statistical determination of the internalization score. Panel C: bright field, fluorescent and overlay images for 9 individual cells representative of the 5 000 cells analyzed in panel B.
(TIF)

S1 Data (XLSX)
