# Supplementary figures and images for: Complementary epitopes and favorable developability of monoclonal anti-LAMP1 antibodies generated using two transgenic animal platforms
Source: PLoS One. 2020 Jul 16;15(7):e0235815. doi: 10.1371/journal.pone.0235815 (PMC7365404; doi:10.1371/journal.pone.0235815)

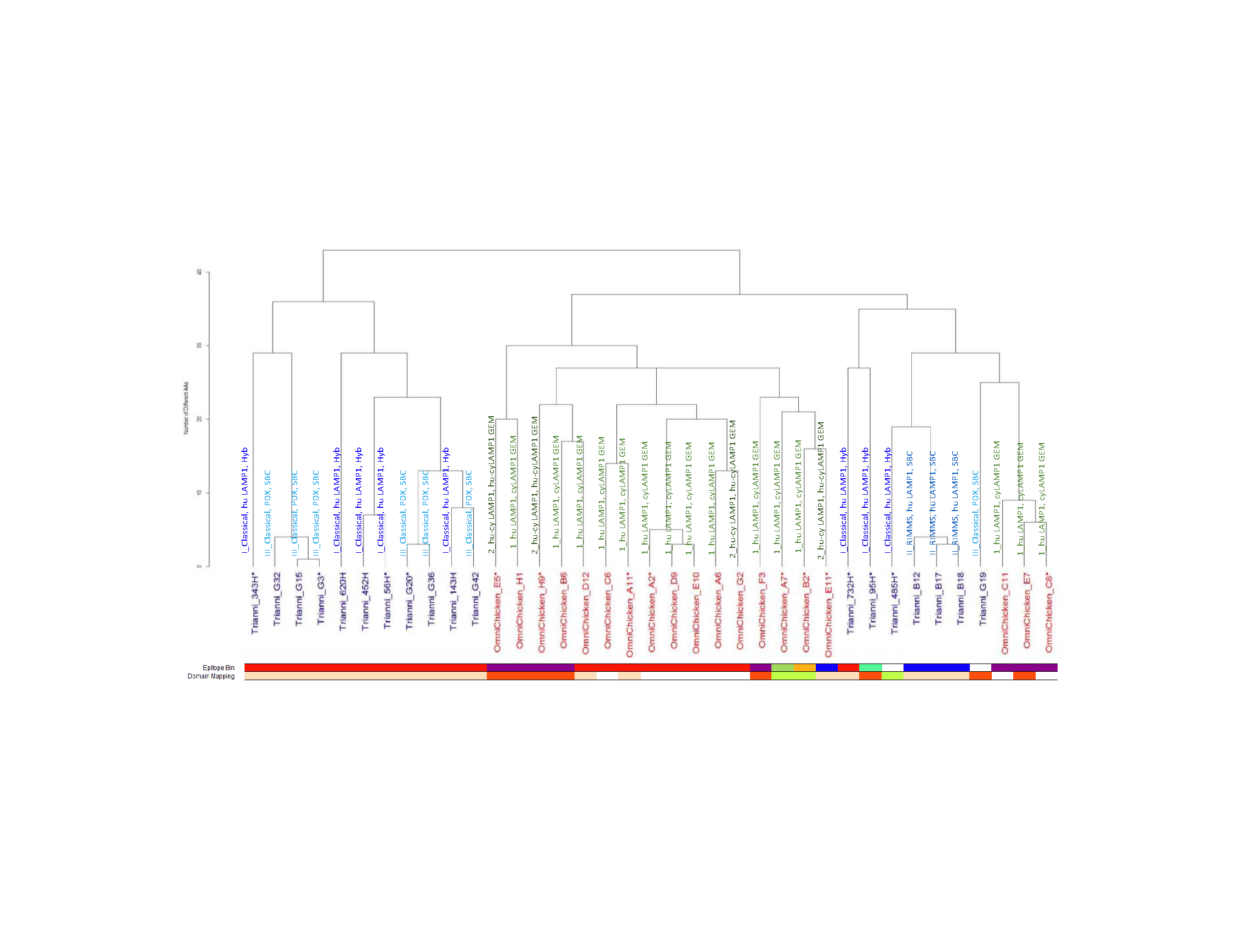

Supplement: S1 Fig — Paratope sequence tree as in Fig 2 with the origin—method of immunization (classical (I, III) or RIMMS (II) for TRIANNI-derived mAbs), antigen (recombinant human LAMP1 (I, II) or PDX (III) for TRIANNI-derived mAbs and only recombinant human- (1), or both human and cynomolgus LAMP1 (2) for OmniChicken-derived mAbs) and selection (hybridoma (I) or single B cell sorting (II, III) for TRIANNI-derived mAbs, and GEM assay with only beads coated with cynomolgus LAMP1 (1) or with both beads coated with human- and beads coated with cynomolgus LAMP1 (2) for OmniChicken-derived mAbs)—of each of the 37 antibodies indicated. (TIFF) [file pone.0235815.s001.tiff]

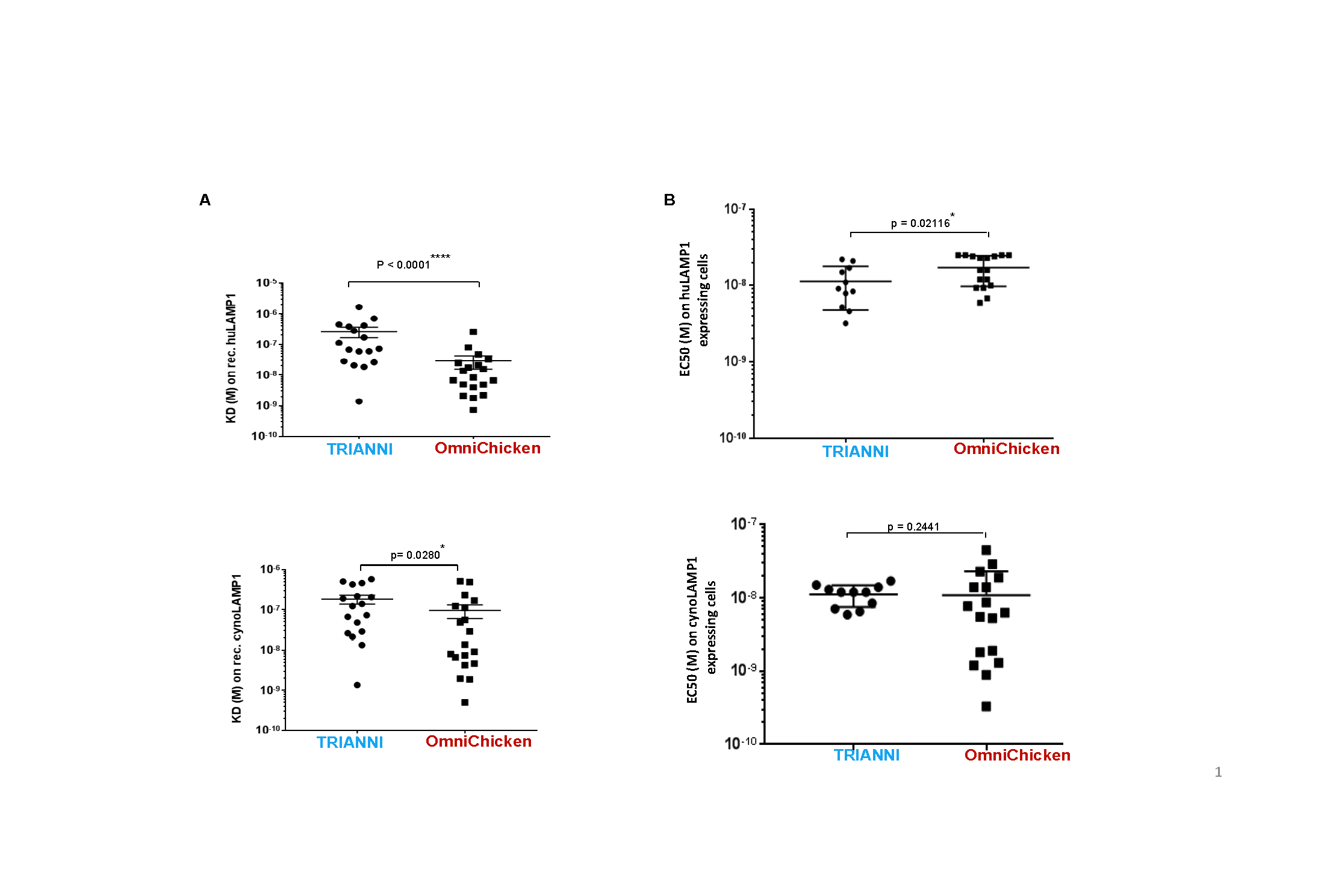

Supplement: S2 Fig — Horizontal lines indicate means ± SEM. (TIFF) [file pone.0235815.s002.tiff]

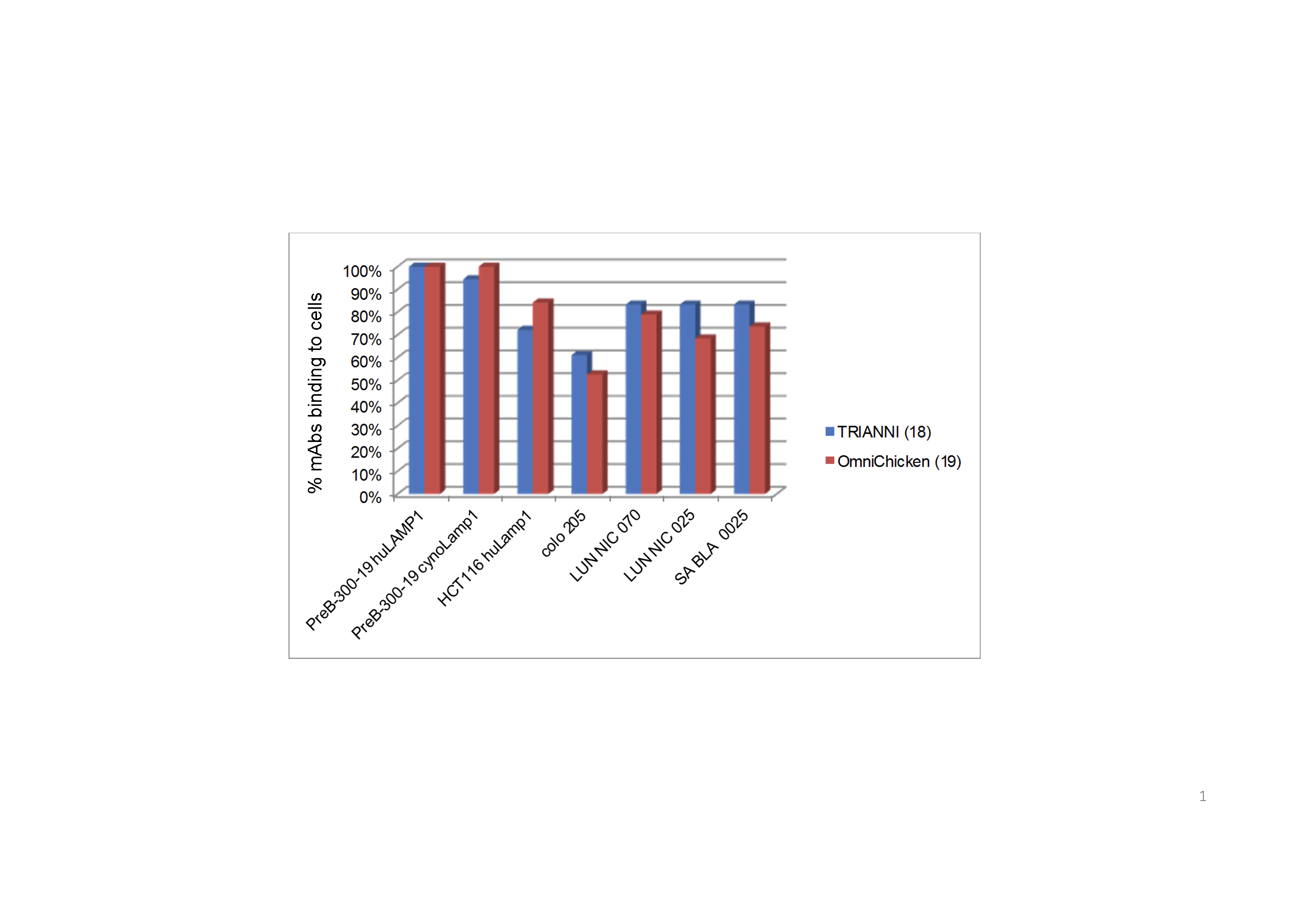

Supplement: S3 Fig — Percentage of TRIANNI- and OmniChicken-derived anti-LAMP1 mAbs binding to the indicated tumor cell line or PDX as determined by flow cytometry. (TIFF) [file pone.0235815.s003.tiff]

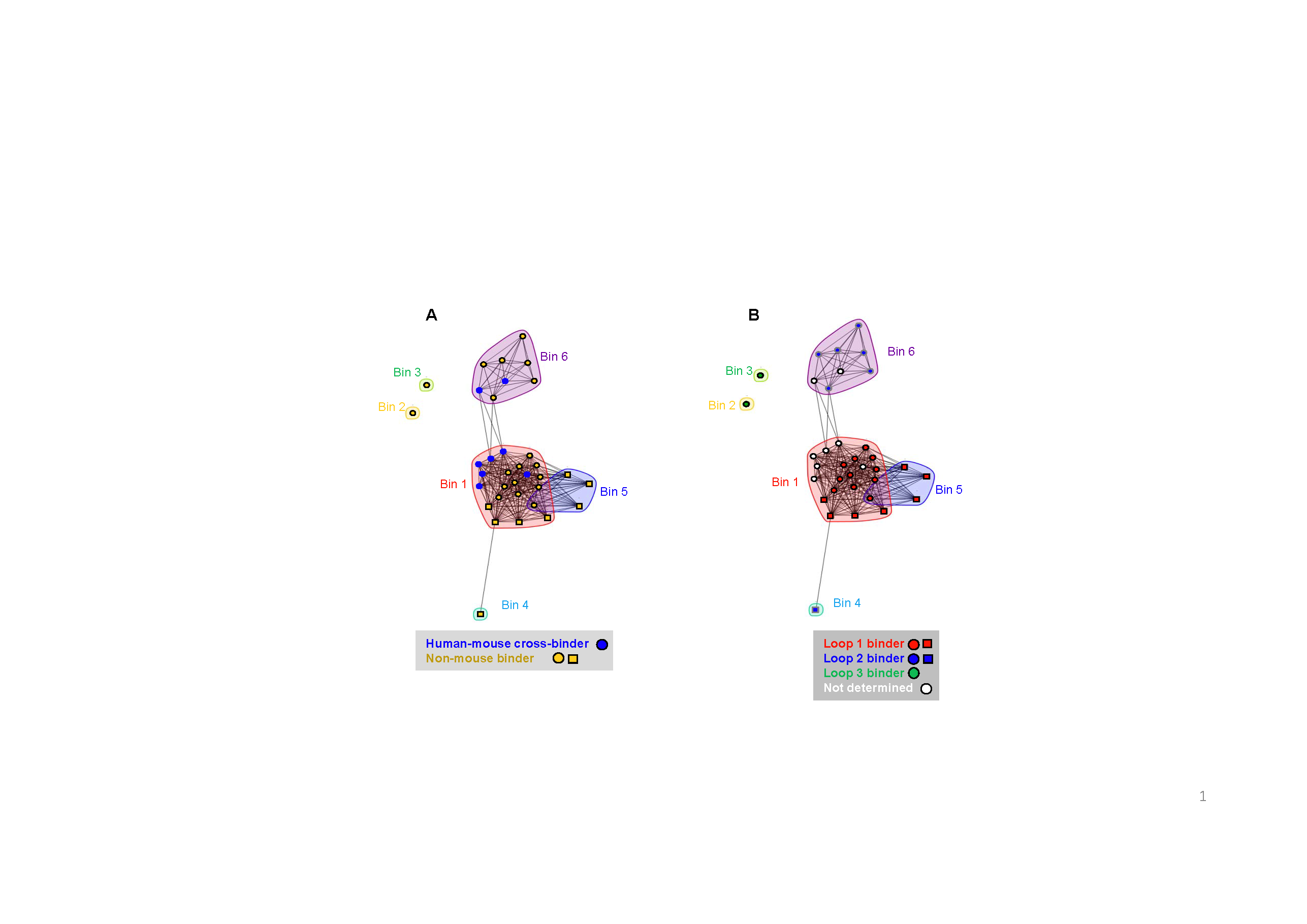

Supplement: S4 Fig — (TIFF) [file pone.0235815.s004.tiff]

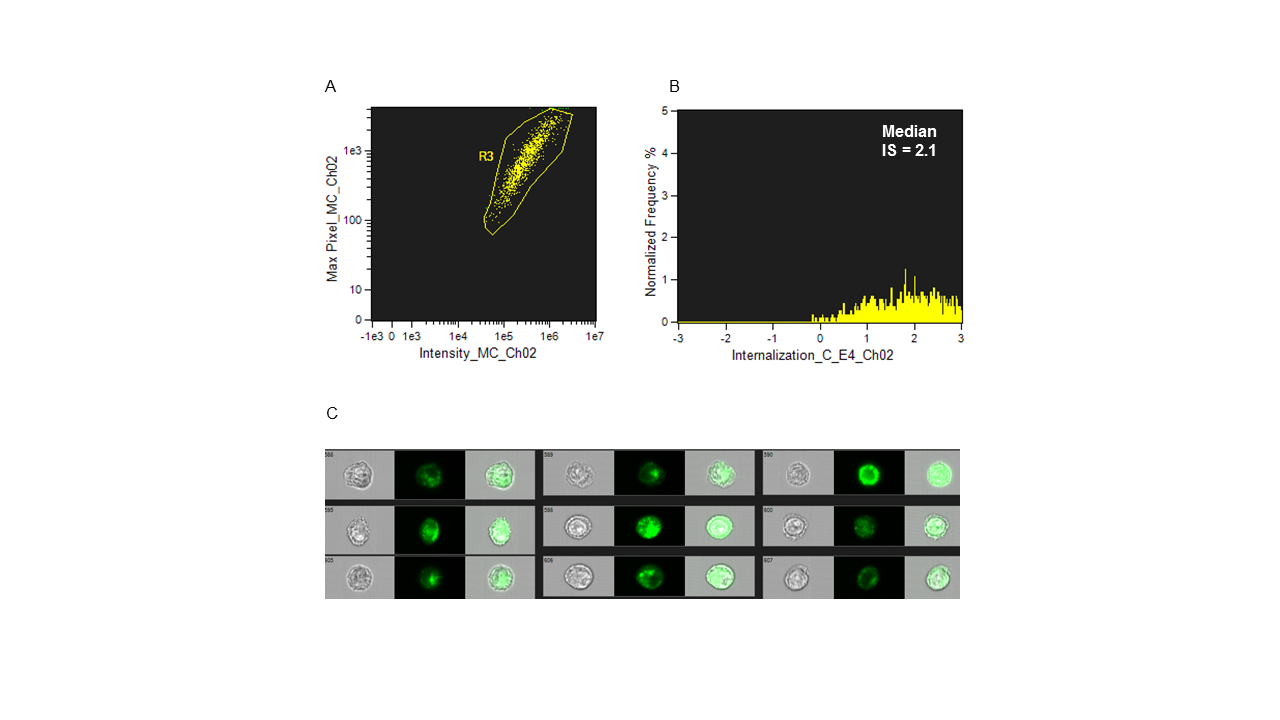

Supplement: S5 Fig — Representative data with labeled TRIANNI _G3 anti-LAMP1 onto LAMP1- expressing HCT116 cells. Panel A: Gating for high Max Pixel and Intensity for at least 5 000 labeled cells. Panel B: Data acquisition on the gated cells for statistical determination of the internalization score. Panel C: bright field, fluorescent and overlay images for 9 individual cells representative of the 5 000 cells analyzed in panel B. (TIF) [file pone.0235815.s005.tif]
